# sAPPα Inhibits Neurite Outgrowth in Primary Mouse Neurons via GABA B Receptor Subunit 1a

**DOI:** 10.1523/ENEURO.0345-25.2026

**Published:** 2026-02-12

**Authors:** Dylan Barber, Casandra Salinas-Salinas, Samah Houmam, Kriti Shukla, Heather C. Rice

**Affiliations:** ^1^Aging & Metabolism Research Program, Oklahoma Medical Research Foundation, Oklahoma City, Oklahoma 73104; ^2^Neuroscience Program, University of Oklahoma Health Sciences Center, Oklahoma City, Oklahoma 73104; ^3^Center for Geroscience and Healthy Brain Aging, University of Oklahoma Health Sciences Center, Oklahoma City, Oklahoma 73104; ^4^Department of Biochemistry & Physiology, University of Oklahoma Health Sciences Center, Oklahoma City, Oklahoma 73104; ^5^Department of Chemistry & Biochemistry, University of Oklahoma, Norman, Oklahoma 73019

**Keywords:** amyloid precursor protein, GABA receptor, mouse primary neurons, neurite outgrowth

## Abstract

Neurite outgrowth is essential for neural circuit formation and is tightly regulated by secreted factors and their receptors. The secreted extracellular domain of the amyloid precursor protein (sAPPα) has been shown to modulate neurite outgrowth. Recently, the gamma amino butyric acid receptor type-B subunit 1a (GABA_B_R1a) was identified as an sAPPα binding partner that mediates its effects on synaptic transmission. Here, we investigated whether this interaction also regulates neurite outgrowth. In mouse primary hippocampal neurons of either sex, the GABA_B_R agonist baclofen reduced axon length; whereas its antagonist CGP54626 increased axon length in primary hippocampal neurons. Moreover, GABA_B_R1a knock-out increased axon length and abolished the effect of baclofen. Application of sAPPα reduced axon length, an effect that required the presence of both GABA_B_R1a and the extension domain of sAPPα, which mediates its binding to GABA_B_R1a. Similarly, the APP 17mer peptide, which is sufficient to bind GABA_B_R1a and mimic the effects of sAPP on synaptic transmission, reduced axon outgrowth in wild-type but not in GABA_B_R1a-deficient neurons. Together, these findings indicate that the 1a isoform contributes to GABA_B_R-dependent suppression of neurite outgrowth and mediates the inhibitory effect of sAPPα on neurite outgrowth.

## Significance Statement

Amyloid precursor protein (APP) plays a central role in Alzheimer's disease, yet its normal functions are not fully understood. In this study, we uncover a previously unrecognized role of the GABA B Receptor in mediating the inhibitory effects of sAPPα on neurite outgrowth. These findings provide mechanistic insight into how disruptions in APP signaling could influence both normal brain development and pathological processes in neurodevelopmental disorders and Alzheimer's disease.

## Introduction

Neurite outgrowth, the process by which neurons extend their axons and dendrites, is essential for establishing neural circuity in the brain. This developmental process is tightly regulated by secreted factors and their receptors. APP is a type I transmembrane protein that undergoes sequential proteolytic processing to generate several fragments, most notably the amyloid-beta (Aβ) peptide which accumulates in Alzheimer's disease (AD; Glenner and Wong, 1984). APP processing also leads to the secretion of other physiologically relevant fragments besides Aβ. The initial cleavage of APP by either α-, β-, or η-secretase releases a large extracellular fragment termed secreted APP (sAPPα, sAPPβ, or sAPPη, respectively; Hefter et al., 2020). During early development, APP expression rises (Hung et al., 1992; Kirazov et al., 2001) and contributes to neurodevelopmental processes such as neurite outgrowth (Nicolas and Hassan, 2014; Chau et al., 2023). Decades of research have shown that primary neurons cultured from APP knock-out mice exhibit enhanced neurite outgrowth (Perez et al., 1997; Young-Pearse et al., 2008; Billnitzer et al., 2013; Liu et al., 2021) and also indicates that sAPPα is an important fragment in mediating this function of APP in neurite outgrowth (Milward et al., 1992; Young-Pearse et al., 2008; Chasseigneaux et al., 2011; Billnitzer et al., 2013; Hasebe et al., 2013; Dorard et al., 2018).

sAPPα interacts with the gamma-aminobutyric acid receptor type B (GABA_B_R; Schwenk et al., 2016; Dinamarca et al., 2019; Rice et al., 2019; Rem et al., 2023), a metabotropic receptor for the inhibitory neurotransmitter GABA. GABA_B_R is an obligate heterodimer composed of two subunits. Subunit 1 binds GABA; subunit 2 couples intracellularly to Guanine nucleotide-binding (G) proteins (Pin and Bettler, 2016). Subunit 1 exists in two main isoforms, with the 1a isoform containing two additional N-terminal sushi domains that are absent in the 1b isoform (Pin and Bettler, 2016). The extension domain (ExD) within the extracellular region of APP was found to bind specifically to the first sushi domain of the 1a isoform (GABABR1a; Rice et al., 2019). GABA_B_R1a was shown to mediate effects of sAPPα on synaptic transmission, and a synthetic 17 amino acid peptide within the ExD of sAPPα (APP 17mer) was sufficient to bind GABA_B_R1a and mimic these effects (Rice et al., 2019). However, whether GABA_B_R1a also mediates other functions of sAPPα is not yet known.

The protein expression of both GABA_B_R and APP rise during the first few weeks of rodent development (Hung et al., 1992; Fritschy et al., 1999; Kirazov et al., 2001; Schwenk et al., 2016; Khoshdel-Sarkarizi et al., 2019). Moreover, both APP and GABA_B_R have been implicated independently in the regulation of similar neurodevelopmental processes, including neurogenesis (Ma et al., 2008; Demars et al., 2011; Giachino et al., 2014), neuronal migration (Young-Pearse et al., 2007; Rice et al., 2012; Bony et al., 2013; Callahan et al., 2017), synaptogenesis (Meier et al., 2008; Fiorentino et al., 2009; Tyan et al., 2012), and neurite outgrowth (Milward et al., 1992; Perez et al., 1997; Gakhar-Koppole et al., 2008; Osterfield et al., 2008; Young-Pearse et al., 2008; Hoe et al., 2009; Sernagor et al., 2010; Chasseigneaux et al., 2011; Billnitzer et al., 2013; Bony et al., 2013; Hasebe et al., 2013; Favuzzi et al., 2021). Here, we sought to determine whether GABA_B_R mediates effects of sAPPα on neurite outgrowth.

Our study demonstrates that knock-out of GABA_B_R1a promotes axon outgrowth in primary hippocampal neurons. We also show that both sAPPα and APP 17mer inhibit axon outgrowth and that these effects can be reversed by either removing the ExD of sAPPα or by genetically ablating GABA_B_R1a. Together, these findings indicate that GABA_B_R1a is a key mediator of the inhibitory effect of sAPPα on axon outgrowth.

## Materials and Methods

### Mouse models

All animal procedures were performed in accordance with the Oklahoma Medical Research Foundation animal care committee's regulations. C57BL/6J mice (Jackson Laboratories) were group housed in the AALAS-accredited OMRF vivarium operating on a 14/10 h light/dark cycle with *ad libitum* access to food and water. Timed mated females and their embryonic litters used to generate primary neurons for experimentation. GABA_B_R1a knock-out (KO) mice generated by the VIB-KU Leuven Center for Brain & Disease Research Mouse Expertise Unit with support from VIB Discovery Sciences. Sperm from GABA_B_R1a KO mice were provided by Joris de Wit, VIB-KU Leuven Center for Brain & Disease Research, Leuven, Belgium, and then mice were rederived at the Texas A&M Institute for Genomic Medicine. For the generation of embryonic litters from GABA_B_R1a KO mice, two heterozygous adults were paired for timed mating. The resulting litters contained WT (+/+), Het (+/−), and KO (−/−) embryos, which were used for primary cultures. Genotyping was performed on utilizing the KAPA Hotstart Mouse Genotyping Kit (Kapa Biosystems KK7352) with the following primers: mGABBR1Fwd1 [5′-GGAAGAAGAACAGGGGGA-3′], mGABBR1Rev1 [5′-AGGAGGTCAGGAGTTGTG-3′].

### Primary mouse hippocampal neuron culture

Hippocampal neurons were isolated from embryonic day 18 (E18) mouse brains of either sex. Hippocampi were dissected by decapitating the embryos and placing the heads into ice-cold Hanks balanced salt solution without magnesium and calcium (Invitrogen 14-175-095), supplemented with the following (in mM): 2.5 HEPES, 30 d-glucose, 1 CaCl_2_, 1 MgSO_4_, and 4 NaHCO_3_ (cHBSS). The brain was extracted, and the hippocampus was dissected into ice-cold cHBSS. Tissues were dissociated by incubation in cHBSS with 0.25% trypsin (Invitrogen 15090046) and DNAse (50 µg/ml; Sigma-Aldrich 11284932001) for 15 min at 37°C, followed by gentle trituration using a flame-polished Pasteur pipette. Cells were washed with cHBSS three times and plated at 75,000 cells/ml on 12 mm coverslips precoated with poly-d-lysine (Neuvitro GG-12-PDL), coated with laminin (0.001 mg/ml; Invitrogen 114956-81-9 L2020). Coverslips were placed into a 12-well cell culture dish (Corning 353043) filled with cHBSS. Neurons were fed by half media change after 3 h with Hippocampal Feeding Media (HFM): Neurobasal medium (Invitrogen 21103049) supplemented with 1× B27 (Invitrogen 17504044), 0.25× GlutaMAX (Invitrogen 35050061), 0.24% d-glucose (Thermo Fisher Scientific A16828.0C), penicillin/streptomycin (20 U/ml; Invitrogen 15140122), and 24.2 µM β-mercaptoethanol (Thermo Fisher Scientific 2198502). For the litters from the GABA_B_R1a^+/−^ matings, embryonic tissue samples were used for genotyping as described above. The dissected hippocampi were dissociated separately and plated onto batches of coverslips independently. After same-day genotyping, coverslips of the three possible genotypes GABA_B_R1a^+/+^, GABA_B_R1a^+/−^, and GABA_B_R1a^−/−^ were selected for treatment. Cells were treated with purified proteins, synthetic peptides, or pharmacological agents by bath application 3 h after plating. Purified sAPPα and sAPPαΔExD proteins (see plasmids and purification methods below) or synthetic APP 17mer and scrambled 17mer peptides (described below) were applied at a final concentration ranging from 1 to 500 nM. The GABA_B_R agonist baclofen (Sigma-Aldrich 63701-55-3) and antagonist (Sigma-Aldrich SML3136) were both bath applied at 10 µM. Neurons were maintained in HFM and fixed 72 h after plating.

### Plasmids

sAPP-Fc constructs were provided by Dr. Joris de Wit, VIB-KU Leuven Center for Brain & Disease Research, Leuven, Belgium. sAPP-Fc constructs were originally generated by PCR-amplifying the following regions of mouse APP695: sAPPα, 18–612 aa; sAPPαΔExD, 19–194 aa and 228–596 aa. Each of the PCR fragments were subcloned between and in frame with the prolactin signal peptide and human Fc in the pLP-FLAG-IgG vector using Gibson Assembly (NEB).

### Protein purification

Secreted C-terminally Fc-tagged proteins were expressed by transient transfection using polyethylenimine (PEI; Kyfora Bio 23966) in HEK293T cells and collected in serum-free Opti-MEM (Invitrogen 31985088). Conditioned medium was passed through a Protein-G Sepharose packed column (Cytiva 17061802) at 4°C, washed with 250 ml of wash buffer (50 mM Tris, pH 8.0, 450 mM NaCl, 1 mM EDTA), and Fc tag cleaved O/N with GST-tagged 3C PreScission Protease (Cytiva 27084301) in cleavage buffer (50 mM Tris, pH 8.0, 150 mM NaCl, 1 mM EDTA, 1 mM DTT). Cleaved protein was collected in the eluate and the protease separated from the eluted proteins using a Glutathione Sepharose (Cytiva 17513201) column collecting in TNE buffer (50 mM Tris, pH 8.0, 150 mM NaCl, 1 mM EDTA). Proteins were dialyzed against phosphate-buffered saline (PBS) O/N, concentrated using centrifugal filter units (Millipore Sigma UFC9010), and depleted of endotoxin with Pierce High Capacity Endotoxin Removal Spin Columns (Thermo Fisher Scientific 88274). Protein concentration was determined by BCA Protein Assay (Thermo Fisher Scientific 23227) and verified by Coomassie SDS-PAGE.

### Western blotting

Lysates obtained from DIV 3 hippocampal primary neurons, HEK293T cells transfected with mouse GABA_B_R1b (pCMV6-Entry vector) or human GABA_B_R1a (pEZ-MO2 vector), and synaptosome extracted from mouse brains were quantified using the BCA protein assay (Thermo Fisher Scientific 23227). Then, 8 µg of primary neuron and synaptosome lysate and 0.5 µg of HEK293 cell lysate was mixed with 10× reducing agent (Invitrogen, NP0004) and 4× NuPAGE LDS Sample Buffer (Invitrogen, NP0007), heated for 8 min at 70°C, and resolved on 4–12% bis-tris Nu-PAGE gels (Invitrogen, NP0322) with MOPS running buffer (Invitrogen, NP0001) in an Invitrogen XCell4 SureLock Midi-Cell (Invitrogen, WR0100). Proteins were transferred onto nitrocellulose membranes (Bio-Rad, 1620112) using Bio-Rad Criterion Western Blot transfer electrophoresis tank (BIORAD, 1704070) at 400 mA for 90 min. Membranes were blocked with 5% BSA in water for 1 h at room temperature and then incubated overnight at 4°C with primary antibodies GBR1 (rabbit, Cell Signaling 3835), β-actin (mouse, Sigma A2228) diluted 1:1,000 in 0.05% Tween-20 in PBS (PBST). After washing three times with PBST, membranes were incubated with secondary antibodies (LICOR, 92632213 and LICOR, 92668072) for 1 h at room temperature. Protein bands were visualized using a fluorescence imaging system (LICOR CLx-9140).

### Synthetic peptides

The following peptides were synthesized by Insight Biotechnology at >98% purity:

APP 17mer (204–220AA of APP695): acetyl-DDSDVWWGGADTDYADG-amide

Scrambled 17mer: acetyl-DWGADTVSGDGYDAWDD-amide.

### Immunocytochemistry and light microscopy

For fluorescence staining, cells were fixed with 4% PFA in PBS for 15 min. Fixed samples were washed three times in PBS, 5 min per wash. The fixed neurons were then blocked with 3% bovine serum albumin in PBS supplemented with Triton X-100 (0.2%) O/N at 4°C and stained with primary mouse monoclonal Tau-1 antibody to an axon specific microtubule associated protein (1:1,000 dilution; Millipore Sigma MAB3420), primary chicken MAP2 antibody to stain dendrites (1:1,000; Abcam Ab5392), and a primary rabbit Tuj1 antibody against pan-neuronal β3 tubulin (1:1,000 dilution; Abcam Ab18207). Samples were then secondary antibody conjugated to Alexa Fluor 488 (Southern Biotech 6410-30), Alexa Fluor 568 (Thermo Fisher Scientific A78950), or Alexa Fluor 647 (BioLegend 406414) reconstituted at 1, 2, and 0.5 mg/ml, respectively, all used at 1:200 dilution. Light microscopy imaging was performed using an Axioscan7 Whole Slide Scanner at 40× air magnification with the OMRF Imaging Core Facility.

### Image analysis

All analysis was performed blinded to treatment conditions. Blinding was achieved through basic Caesar cipher of files names and pseudo labeling of slides prior to imaging on the Axioscan 7. The region of interest for analysis was 21 mm^2^ and neurons were measured within the ROI in clockwise manner starting from a random corner of the image until the target number of neurons were measured (60–90/coverslip). Measurements were taken using ImageJ Simple Neurite Tracer (SNT) software. Axon lengths were measured from the axon hillock to the tip of the longest primary neurite stained by Tau-1 (Axon Marker). Neurite lengths were determined in three independent experiments using images acquired at 25–40× magnification stained by Tau-1 (Axon Marker) and MAP2 (Dendrite Marker) for the purpose of differentiating the neuronal compartments. Images were imported into ImageJ using the bio-formats importer plug-in. The images were then imported into the SNT plug-in and axon filaments were traced using the filament tracer algorithm. Blinding was removed after datasets were collected. Neuronal polarity was determined by categorizing neurons possessing multiple axons (two or more neurites stained predominantly by Tau-1), a single axon (one neurite stained predominantly by Tau-1), or no axon (no neurites stained predominantly by Tau-1).

### Statistical analysis

Statistical analysis was performed using GraphPad Prism software and is summarized in Table 1. Normality was determined by Anderson–Darling and Shapiro–Wilk test. Significance was determined using Kruskal–Wallis with Dunn's multiple-comparison post hoc test. Graphs were produced using GraphPad Prism.

**Table 1.** Statistical table

Figure	Line	Data structure	Test	Post hoc test	*p*	Power (95% C.I. of diff, lower)	Power (95% C.I. of diff, upper)
1*B*: +/+; Control	224	Non-normal distribution	Kruskal–Wallis	Dunn's			
1*B*: +/+; Baclofen	224	Non-normal distribution	Kruskal–Wallis	Dunn's	<0.0001	0.1041	0.096
1*B*: +/+; CGP	224	Non-normal distribution	Kruskal–Wallis	Dunn's	<0.0001	−0.102	−0.114
1*E*: +/+	232	Non-normal distribution	Kruskal–Wallis	Dunn's			
1*E*: +/−	232	Non-normal distribution	Kruskal–Wallis	Dunn's	<0.0001	−0.37	−0.468
1*E*: −/−	232	Non-normal distribution	Kruskal–Wallis	Dunn's	0.0016	−0.127	−0.175
1*F*, 3*B*: +/+; Control	237	Non-normal distribution	Kruskal–Wallis	Dunn's			
1*F*, 3*B*: +/−; Control	237	Non-normal distribution	Kruskal–Wallis	Dunn's			
1*F*, 3*B*: −/−; Control	237	Non-normal distribution	Kruskal–Wallis	Dunn's			
1*F*: +/+; Baclofen	237	Non-normal distribution	Kruskal–Wallis	Dunn's	<0.0001	0.3767	0.4209
1*F*: +/−; Baclofen	237	Non-normal distribution	Kruskal–Wallis	Dunn's	<0.0001	0.3797	0.3924
1*F*: −/−; Baclofen	237	Non-normal distribution	Kruskal–Wallis	Dunn's	0.6234	0.071	0.072
1-1: +/+	233	Non-normal distribution	Kruskal–Wallis	Dunn's			
1-1: +/−	233	Non-normal distribution	Kruskal–Wallis	Dunn's	0.0808	1.261	1.739
1-1: −/−	233	Non-normal distribution	Kruskal–Wallis	Dunn's	0.0281	1.473	1.681
1-2, 3-1: +/+; Control	239	Normal distribution	Brown–Forsythe and Welch	Dunnett T3			
1-2, 3-1: +/−; Control	239	Normal distribution	Brown–Forsythe and Welch	Dunnett T3			
1-2, 3-1: −/−; Control	239	Normal distribution	Brown–Forsythe and Welch	Dunnett T3			
1-2: +/+; Baclofen	239	Normal distribution	Brown–Forsythe and Welch	Dunnett T3	0.4407	0.942	1.378
1-2: +/−; Baclofen	239	Normal distribution	Brown–Forsythe and Welch	Dunnett T3	>0.9999	0.135	−1.447
1-2: −/−; Baclofen	239	Normal distribution	Brown–Forsythe and Welch	Dunnett T3	>0.9999	−0.45	−0.576
1-3: +/+; Control MA	242	Normal distribution	Brown–Forsythe and Welch	Dunnett T3			
1-3: +/−; Control MA	242	Normal distribution	Brown–Forsythe and Welch	Dunnett T3			
1-3: −/−; Control MA	242	Normal distribution	Brown–Forsythe and Welch	Dunnett T3			
1-3: +/+; Baclofen MA	242	Normal distribution	Brown–Forsythe and Welch	Dunnett T3	0.8114	−0.00132	−0.05865
1-3: +/−; Baclofen MA	242	Normal distribution	Brown–Forsythe and Welch	Dunnett T3	>0.9999	−0.014068	0.0607
1-3: −/−; Baclofen MA	242	Normal distribution	Brown–Forsythe and Welch	Dunnett T3	>0.9999	−0.060925	0.0609
1-3: +/+; Control SA	242	Normal distribution	Brown–Forsythe and Welch	Dunnett T3			
1-3: +/−; Control SA	242	Normal distribution	Brown–Forsythe and Welch	Dunnett T3			
1-3: −/−; Control SA	242	Normal distribution	Brown–Forsythe and Welch	Dunnett T3			
1-3: +/+; Baclofen SA	242	Normal distribution	Brown–Forsythe and Welch	Dunnett T3	>0.9999	−0.088	−0.0186
1-3: +/−; Baclofen SA	242	Normal distribution	Brown–Forsythe and Welch	Dunnett T3	>0.9999	−0.0953	0.1819
1-3: −/−; Baclofen SA	242	Normal distribution	Brown–Forsythe and Welch	Dunnett T3	>0.9999	−0.2558	0.2892
1-3: +/+; Control NA	242	Normal distribution	Brown–Forsythe and Welch	Dunnett T3			
1-3: +/−; Control NA	242	Normal distribution	Brown–Forsythe and Welch	Dunnett T3			
1-3: −/−; Control NA	242	Normal distribution	Brown–Forsythe and Welch	Dunnett T3			
1-3: +/+; Baclofen NA	242	Normal distribution	Brown–Forsythe and Welch	Dunnett T3	>0.9999	0.03468	0.1321
1-3: +/−; Baclofen NA	242	Normal distribution	Brown–Forsythe and Welch	Dunnett T3	0.9546	−0.17707	0.0503
1-3: −/−; Baclofen NA	242	Normal distribution	Brown–Forsythe and Welch	Dunnett T3	>0.9999	−0.13248	0.1126
2*D*: +/+; Control	253	Non-normal distribution	Kruskal–Wallis	Dunn's			
2*D*: +/+; sAPPα 1 nM	253	Non-normal distribution	Kruskal–Wallis	Dunn's	<0.0001	−0.214	−0.248
2*D*: +/+; sAPPα 20 nM	253	Non-normal distribution	Kruskal–Wallis	Dunn's	<0.0001	−0.211	−0.214
2*D*: +/+; sAPPα 50 nM	253	Non-normal distribution	Kruskal–Wallis	Dunn's	<0.0001	−0.206	−0.22
2*D*: +/+; sAPPα 200 nM	253	Non-normal distribution	Kruskal–Wallis	Dunn's	0.0018	−0.156	−0.178
2*D*: +/+; sAPPα 500 nM	253	Non-normal distribution	Kruskal–Wallis	Dunn's	<0.0001	0.1904	0.1933
2*F*: +/+; Control	256	Non-normal distribution	Kruskal–Wallis	Dunn's			
2*F*: +/+; sAPPα	256	Non-normal distribution	Kruskal–Wallis	Dunn's	<0.0001	0.3268	0.3721
2*F*:+/+; ΔExD	256	Non-normal distribution	Kruskal–Wallis	Dunn's	>0.9999	−0.048	−0.044
2*F*:+/+; APP 17mer	256	Non-normal distribution	Kruskal–Wallis	Dunn's	0.002	0.0871	0.026
2*F*:+/+; Scrambled 17mer	256	Non-normal distribution	Kruskal–Wallis	Dunn's	>0.9999	0.011	−0.068
3*B*: +/+; sAPPα	270	Non-normal distribution	Kruskal–Wallis	Dunn's	<0.0001	0.281	0.2952
3*B*: +/+; ΔExD	270	Non-normal distribution	Kruskal–Wallis	Dunn's	0.191	−0.085	−0.085
3*B*: +/+; APP 17mer	270	Non-normal distribution	Kruskal–Wallis	Dunn's	<0.0001	0.2901	0.2832
3*B*: +/+; Scrambled 17mer	270	Non-normal distribution	Kruskal–Wallis	Dunn's	0.7126	0.0896	0.117
3*B*: +/−; sAPPα	270	Non-normal distribution	Kruskal–Wallis	Dunn's	<0.0001	0.3315	0.3371
3*B*: +/−; ΔExD	270	Non-normal distribution	Kruskal–Wallis	Dunn's	>0.9999	0.027	0.021
3*B*: +/−; APP 17mer	270	Non-normal distribution	Kruskal–Wallis	Dunn's	<0.0001	0.411	0.4277
3*B*: +/−; Scrambled 17mer	270	Non-normal distribution	Kruskal–Wallis	Dunn's	>0.9999	−0.037	−0.059
3*B*: −/−; sAPPα	270	Non-normal distribution	Kruskal–Wallis	Dunn's	>0.9999	0.034	0.071
3*B*: −/−; ΔExD	270	Non-normal distribution	Kruskal–Wallis	Dunn's	>0.9999	0.043	0.083
3*B*: −/−; APP 17mer	270	Non-normal distribution	Kruskal–Wallis	Dunn's	>0.9999	−0.004	−0.007
3*B*: −/−; Scrambled 17mer	270	Non-normal distribution	Kruskal–Wallis	Dunn's	0.3425	−0.054	−0.047
3-1: +/+; sAPPα	275	Non-normal distribution	Kruskal–Wallis	Dunn's	>0.9999	−0.143	−0.092
3-1: +/+; ΔExD	275	Non-normal distribution	Kruskal–Wallis	Dunn's	0.9187	0.704	0.773
3-1: +/+; APP 17mer	275	Non-normal distribution	Kruskal–Wallis	Dunn's	0.4921	0.931	1.001
3-1: +/+; Scrambled 17mer	275	Non-normal distribution	Kruskal–Wallis	Dunn's	>0.9999	0.01	0.288
3-1: +/−; sAPPα	275	Non-normal distribution	Kruskal–Wallis	Dunn's	0.3668	−0.981	−1.21
3-1: +/−; ΔExD	275	Non-normal distribution	Kruskal–Wallis	Dunn's	>0.9999	−0.554	−1.289
3-1: +/−; APP 17mer	275	Non-normal distribution	Kruskal–Wallis	Dunn's	>0.9999	−0.319	−1.561
3-1: +/−; Scrambled 17mer	275	Non-normal distribution	Kruskal–Wallis	Dunn's	>0.9999	−0.409	−1.117
3-1: −/−; sAPPα	275	Non-normal distribution	Kruskal–Wallis	Dunn's	>0.9999	−0.198	−0.675
3-1: −/−; ΔExD	275	Non-normal distribution	Kruskal–Wallis	Dunn's	0.0695	−1.309	−1.717
3-1: −/−; APP 17mer	275	Non-normal distribution	Kruskal–Wallis	Dunn's	>0.9999	0.416	0.83
3-1: −/−; Scrambled 17mer	275	Non-normal distribution	Kruskal–Wallis	Dunn's	>0.9999	−0.378	−0.039
3-2: +/+; sAPPα MA	275	Normal distribution	Brown–Forsythe and Welch	Dunnett T3	0.6682	−0.01617	−0.08385
3-2: +/−; sAPPα MA	275	Normal distribution	Brown–Forsythe and Welch	Dunnett T3	0.999	−0.043068	0.1164
3-2: −/−; sAPPα MA	275	Normal distribution	Brown–Forsythe and Welch	Dunnett T3	>0.9999	−0.08443	0.0578
3-2: +/+; sAPPα SA	275	Normal distribution	Brown–Forsythe and Welch	Dunnett T3	>0.9999	0.1729	−0.0595
3-2: +/−; sAPPα SA	275	Normal distribution	Brown–Forsythe and Welch	Dunnett T3	>0.9999	−0.2573	0.2572
3-2: −/−; sAPPα SA	275	Normal distribution	Brown–Forsythe and Welch	Dunnett T3	>0.9999	−0.3211	0.3812
3-2: +/+; sAPPα NA	275	Normal distribution	Brown–Forsythe and Welch	Dunnett T3	0.8795	0.09195	−0.1119
3-2: +/−; sAPPα NA	275	Normal distribution	Brown–Forsythe and Welch	Dunnett T3	>0.9999	−0.16077	0.0941
3-2: −/−; sAPPα NA	275	Normal distribution	Brown–Forsythe and Welch	Dunnett T3	0.9885	−0.25618	0.2163
3-2: +/+; ΔExD MA	275	Normal distribution	Brown–Forsythe and Welch	Dunnett T3	0.952	−0.00698	−0.05305
3-2: +/−; ΔExD MA	275	Normal distribution	Brown–Forsythe and Welch	Dunnett T3	>0.9999	0.012392	0.0476
3-2: −/−; ΔExD MA	275	Normal distribution	Brown–Forsythe and Welch	Dunnett T3	>0.9999	−0.09443	0.0478
3-2: +/+; ΔExD SA	275	Normal distribution	Brown–Forsythe and Welch	Dunnett T3	>0.9999	0.0559	0.0775
3-2: +/−; ΔExD SA	275	Normal distribution	Brown–Forsythe and Welch	Dunnett T3	>0.9999	−0.047	0.0869
3-2: −/−; ΔExD SA	275	Normal distribution	Brown–Forsythe and Welch	Dunnett T3	>0.9999	−0.3276	0.2077
3-2: +/+; ΔExD NA	275	Normal distribution	Brown–Forsythe and Welch	Dunnett T3	>0.9999	−0.0605	−0.0194
3-2: +/−; ΔExD NA	275	Normal distribution	Brown–Forsythe and Welch	Dunnett T3	>0.9999	−0.06302	−0.0303
3-2: −/−; ΔExD NA	275	Normal distribution	Brown–Forsythe and Welch	Dunnett T3	0.9999	−0.11501	0.2817
3-2: +/+; APP 17mer MA	275	Normal distribution	Brown–Forsythe and Welch	Dunnett T3	0.866	0.03871	−0.13875
3-2: +/−; APP 17mer MA	275	Normal distribution	Brown–Forsythe and Welch	Dunnett T3	>0.9999	−0.011878	0.0718
3-2: −/−; APP 17mer MA	275	Normal distribution	Brown–Forsythe and Welch	Dunnett T3	>0.9999	−0.09093	0.0309
3-2: +/+; APP 17mer SA	275	Normal distribution	Brown–Forsythe and Welch	Dunnett T3	>0.9999	−0.036	0.1361
3-2: +/−; APP 17mer SA	275	Normal distribution	Brown–Forsythe and Welch	Dunnett T3	>0.9999	−0.1042	0.1774
3-2: −/−; APP 17mer SA	275	Normal distribution	Brown–Forsythe and Welch	Dunnett T3	>0.9999	−0.2922	0.2589
3-2: +/+; APP 17mer NA	275	Normal distribution	Brown–Forsythe and Welch	Dunnett T3	>0.9999	0.0101	−0.0167
3-2: +/−; APP 17mer NA	275	Normal distribution	Brown–Forsythe and Welch	Dunnett T3	0.9839	−0.16287	0.0429
3-2: −/−; APP 17mer NA	275	Normal distribution	Brown–Forsythe and Welch	Dunnett T3	0.9998	−0.20158	0.2883
3-2: +/+; Scrambled 17mer MA	275	Normal distribution	Brown–Forsythe and Welch	Dunnett T3	0.592	0.032504	−0.05915
3-2: +/−; Scrambled 17mer MA	275	Normal distribution	Brown–Forsythe and Welch	Dunnett T3	0.9998	−0.046408	0.113
3-2: −/−; Scrambled 17mer MA	275	Normal distribution	Brown–Forsythe and Welch	Dunnett T3	>0.9999	−0.05078	0.0241
3-2: +/+; Scrambled 17mer SA	275	Normal distribution	Brown–Forsythe and Welch	Dunnett T3	0.9854	0.1077	−0.0677
3-2: +/−; Scrambled 17mer SA	275	Normal distribution	Brown–Forsythe and Welch	Dunnett T3	>0.9999	−0.1557	0.0422
3-2: −/−; Scrambled 17mer SA	275	Normal distribution	Brown–Forsythe and Welch	Dunnett T3	0.9977	−0.2691	0.2759
3-2: +/+; Scrambled 17mer NA	275	Normal distribution	Brown–Forsythe and Welch	Dunnett T3	0.5878	0.08592	−0.0992
3-2: +/−; Scrambled 17mer NA	275	Normal distribution	Brown–Forsythe and Welch	Dunnett T3	>0.9999	0.00141	0.0586
3-2: −/−; Scrambled 17mer NA	275	Normal distribution	Brown–Forsythe and Welch	Dunnett T3	0.8738	−0.20008	0.2268

## Results

**Figure 1.**
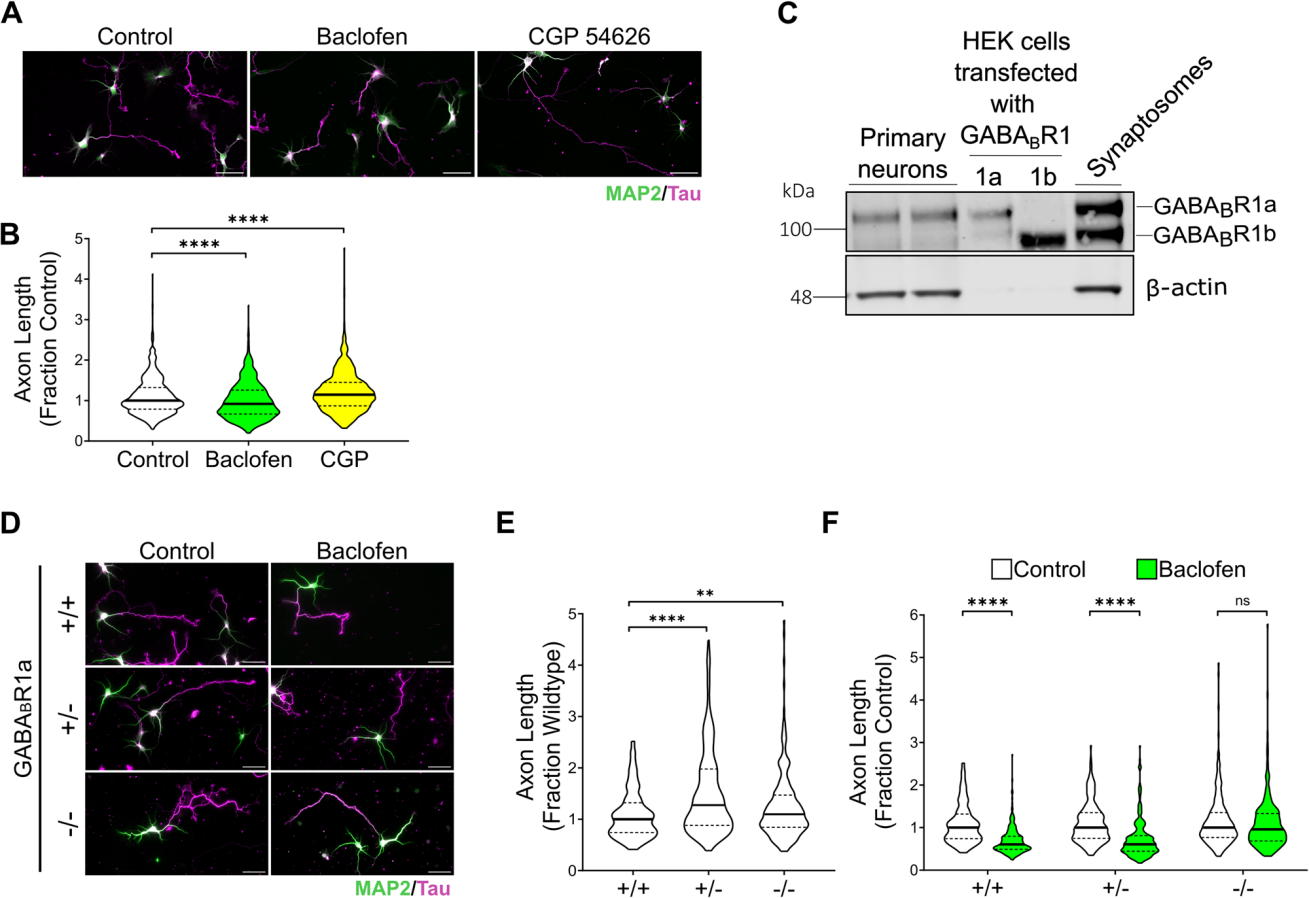
Modulation of GABA_B_R influences axon outgrowth in primary neurons. ***A***, Representative images of hippocampal primary neurons derived from C57BL/6J E18 mouse pups treated at DIV 0 with baclofen (GABA_B_R agonist) or CGP54626 (GABA_B_R antagonist) and immunostained at DIV3 with MAP2 (green, dendritic marker) and Tau (magenta, axonal marker). ***B***, Baclofen treatment significantly decreased axon length (*N* = 180–220 neurons/trial across 5 trials; median = 0.9187, IQR = 0.6672–1.259). CGP54626 treatment significantly increased axon outgrowth (*N* = 180–220 neurons/trial across 5 trials; median = 1.145, IQR = 0.8696–1.451) compared with untreated controls. ***C***, Western blot of DIV 3 primary cultures probed for GABABR1 and β-actin. HEK293 cells transfected with GABA_B_R1a and GABA_B_R1b as well as synaptosomes are shown for reference. Then, 8 µg of protein was loaded for primary cultures and synaptosomes, whereas only 0.5 was loaded of transfected HEK293 cells. ***D***, Representative images of hippocampal primary neurons derived from wild-type (+/+), heterozygous GABA_B_R1a KO (−/+), and homozygous GABA_B_R1a KO (−/−) E18 littermates treated at DIV 0 with baclofen and immunostained at DIV3 with MAP2 (green, dendritic marker) and Tau (magenta, axonal marker). ***E***, Axon length was significantly increased in both GABA_B_R1a (−/−) and (−/+) neurons compared with controls (*N* = 60–90 neurons/trial across 3 trials; medians = 1.096 and 1.274; IQR = 0.8413–1.469 and 0.8797–1.977, respectively). ***F***, Baclofen treatment significantly decreased axon length in primary neurons derived from GABA_B_R1a (+/+) and (−/+) mice (*N* = 60–90 neurons/trial across 3 trials; medians = 0.6067 and 0.6067, IQR = 0.4911–0.7975 and 0.4461–0.8122, respectively). Controls from panel ***D*** and ***E*** are the same, normalized differently for comparison. In panel ***E***, each genotype is normalized to its own untreated control. Polarity graphs show means and standard deviation. Other graphs show medians and interquartile ranges. Scale bars, 50 µm. Kruskal–Wallis with Dunn's multiple-comparison post hoc test were used. **p < 0.01, ****p < 0.0001; ns, not significant (*p* > 0.05). Axon branch number is quantified in Extended Data Figure 1-1 and neuronal polarity is quantified in Extended Data Figure 1-2.

GABA_B_R has been implicated in the regulation of neurite outgrowth of mouse primary neurons (Bony et al., 2013). To confirm these findings, we treated hippocampal neurons isolated from wild-type C57BL/6J E18 mouse embryos with baclofen, an agonist of GABA_B_R, or CGP54626, an antagonist of GABA_B_R. Neurons were treated 3 h after plating and immunostained after 3 d in vitro (3 DIV) for MAP2 to label dendrites and Tau to label axons (Fig. 1*A*). Consistent with previous findings (Bony et al., 2013), we found that bath application of 10 µM baclofen significantly reduced axon length by 9% compared with untreated controls and 10 µM CGP 54626 increased axon length by 14% (Fig. 1*B*). By Western blot analysis, both GABA_B_R1A and GABA_B_R1B are expressed in these cultures at 3 DIV, with GABA_B_R1A being more prominent (Fig. 1*C*). Baclofen and CGP54626 are not isoform-specific in their modulation of the GABA_B_R; therefore, to determine whether GABA_B_R1a, which specifically binds sAPPα, regulates neurite outgrowth, we performed neurite outgrowth assays on primary neurons isolated from littermates that were full KO (GABA_B_R1a^−/−^), heterozygous KO (GABA_B_R1a^+/−^), or wild-type (GABA_B_R1a^+/+^; Fig. 1*D*). We found that the length of the longest axon was significantly increased by 27% in GABA_B_R1a^+/−^ neurons and 9% in GABA_B_R1a^−/−^ as compared with GABA_B_R1a^+/+^ (Fig. 1*E*). Primary axon branches were reduced in GABA_B_R1a^−/−^ neurons as compared with GABA_B_R1a^+/+^ (Extended Data Fig. 1-1*A*).

10.1523/ENEURO.0345-25.2026.f1-1 Figure 1-1 Full genetic ablation of GABA_B_R1a decreases axonal branch number but is unaffected by baclofen treatment. **A)** The number of primary axon branches was decreased in GABA_B_R1a KO (-/-) primary neurons (N = 15-30 neurons/trial across 3 trials; Median = 2.5, IQR = 1-5) compared to controls. Kruskal-Wallis with Dunn’s multiple comparison post hoc test were used. *P < 0.05; ns, not significant (P > 0.05). **B)** The number of primary axon branches of baclofen treated neurons show no significant differences from untreated controls across all genotypes (+/+),(+/-),(-/-) (N = 15-30 primary axons/trial across 3 trials; Untreated Control Medians = 4.5,3.0,3.0, IQRs = 2.0-7.0, 1.75-4.25, 1.0-5.0 respectively). Kruskal-Wallis with Dunn’s multiple comparison post hoc test were used. Comparisons not displayed are not significant (P > 0.05). Download Figure 1-1, TIF file.

**Figure 2.**
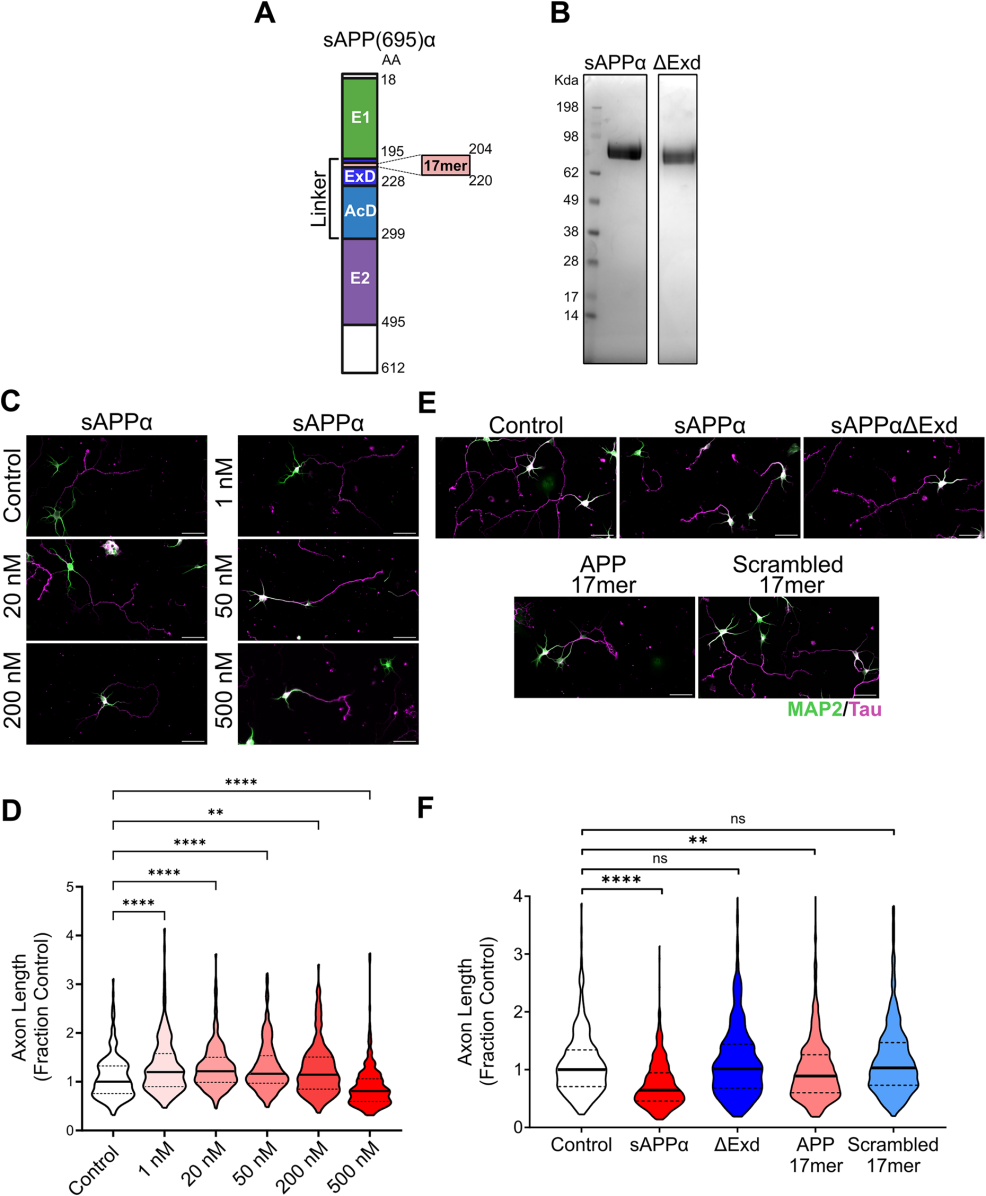
sAPPα reduces axon length in primary neurons in a dose-dependent manner. ***A***, Diagram of the domains of sAPP(695)α. ***B***, Coomassie stain of sAPPα and sAPPα-ΔExD purified proteins. ***C***, Representative images of hippocampal primary neurons derived from C57BL/6J E18 mouse pups treated at DIV 0 with sAPPα at increasing concentrations 1–500 nM. ***D***, Treatment with sAPPα at lower concentrations 1–200 nM increased axon outgrowth compared with controls (*N* = 60–90 neurons/trial across 3 trials; medians = 1.200 and 1.145, IQR = 0.9020–1.579 and 0.8508–1.508, respectively), whereas 500 nM treatment reduced axon outgrowth (*N* = 60–90 neurons/trial across 3 trials; medians = 0.8069, IQR = 0.5983–1.062). ***E***, Representative images of hippocampal primary neurons derived from C57BL/6J E18 mouse pups treated at DIV 0 with sAPPα, sAPPα-ΔExD, APP 17mer, or scrambled 17mer and immunostained at DIV3 with MAP2 (green, dendritic marker) and Tau (magenta, axonal marker). ***F***, Treatment with sAPPα and 17mer decreased axon length in primary neurons compared with untreated controls (*N* = 180–200 neurons/trial across 3 trials; medians = 0.6461 and 0.8894, IQR = 0.4562–0.9462 and 0.6007–1.258, respectively). Treatment with sAPPα-ΔExD and scrambled 17mer had no significant effect on axon length. Graphs show medians and interquartile ranges. Scale bars, 50 µm. Kruskal–Wallis with Dunn's multiple-comparison post hoc test were used. **p < 0.01, ****p < 0.0001; ns, not significant.

10.1523/ENEURO.0345-25.2026.f1-2 Figure 1-2 Neuronal polarity is unaffected by genetic ablation of GABA_B_R1a. Representative images of neurons with multi axon (MA) single axon (SA)and no axon (NA). The fraction of SA, NA, MA neurons across genotypes all (+/+),(+/-),(-/-). Treatment with baclofen was not significantly different from untreated controls (N = 90-160 neurons/trial across 3 trials; Untreated Control Means SA = 0.7167, 0.7300, 0.6700; StDev. = 0.04041, 0.1136, 0.1513; Untreated Control Means NA = 0.2267, 0.1433, 0.1159; StDev = 0.04041, 0.06658, 0.06692; Untreated Control Means MA = 0.05667, 0.1300, 0.06667; StDev = 0.01155, 0.05292, 0.05508 respectively). Comparisons not displayed are not significant (P > 0.05). Download Figure 1-2, TIF file.

To determine the contribution of GABA_B_R1a to the reduction in axonal length by baclofen, we treated GABA_B_R1a^−/−^, GABA_B_R1a^−/+^, and GABA_B_R1a^+/+^ neurons with 10 µM baclofen. Baclofen significantly reduced axon length by 40% in both wild-type and heterozygous GABA_B_R1a KO neurons but produced no significant changes to axon length of GABA_B_R1a KO neurons (Fig. 1*F*). Baclofen produced no significant effects on the number of primary axon branches in any of the three genotypes (Extended Data Fig. 1-1*B*). Since effects on neuronal polarity can lead to changes in neurite outgrowth assays, we quantified the fraction of neurons with a single axon (SA), no axon (NA), or multi-axons (MA) and found that baclofen did not significantly affect polarity with baclofen treatment in any of the genotypes (Extended Data Fig. 1-2). These findings, taken together, demonstrate that GABA_B_R1a reduces axon outgrowth in primary neuron cultures.

sAPPα binds to the sushi-1 domain present in the 1a isoform of GABA_B_R (Rice et al., 2019); therefore, we sought to determine whether GABA_B_R1a mediates the effects of sAPPα on neurite outgrowth. sAPPα (Fig. 2*A*) was affinity purified from transfected–human embryonic kidney (HEK) 293 T cell supernatants (Fig. 2*B*). Previous studies evaluating sAPPα treatment have used concentrations ranging from 1 to 150 nM (Young-Pearse et al., 2008; Billnitzer et al., 2013; Hasebe et al., 2013); however, sAPPα binds the sushi-1 domain at a dissociation constant (K_D_) of 431 nM (Rice et al., 2019). Therefore, we treated neurons with concentrations of sAPPα ranging from 1 to 500 nM. sAPPα at lower concentrations (1–200 nM) increased length of the longest axon between 14 and 21%; whereas 500 nM sAPPα decreased the length of the longest axon by 20% (Fig. 2*D*). To determine if the GABA_B_R1a binding region is required for this inhibitory effect of sAPPα on axon outgrowth, we treated wild-type neurons with 500 nM sAPPα or sAPPα lacking the ExD (sAPPα-ΔExD; Fig. 2*B*). Treatment with 500 nM sAPPα reduced axon length by 36% compared with untreated controls (Fig. 2*E*,*F*). Treatment with 500 nM sAPPα-ΔExD did not significantly affect axon length compared with untreated controls (Fig. 2*E*,*F*), indicating that inhibition of axon outgrowth by sAPPα requires the ExD. Previously, binding between sAPPα and GABA_B_R1a was mapped to a 17aa region within the ExD of APP (Rice et al., 2019). This APP 17mer was sufficient to mimic the GABA_B_R1a-dependent effects on synaptic transmission (Rice et al., 2019). To determine if APP 17mer could also modulate axon outgrowth, we applied 500 nM APP 17mer or its scrambled control peptide. APP 17mer reduced axon length by 11% compared with untreated controls (Fig. 2*E*,*F*); whereas, a scrambled 17mer peptide did not significantly affect axon length. Together, these findings are consistent with a role for GABA_B_R1a in mediating the inhibitory effect of 500 nM sAPPα on neurite outgrowth in primary mouse neurons.

**Figure 3.**
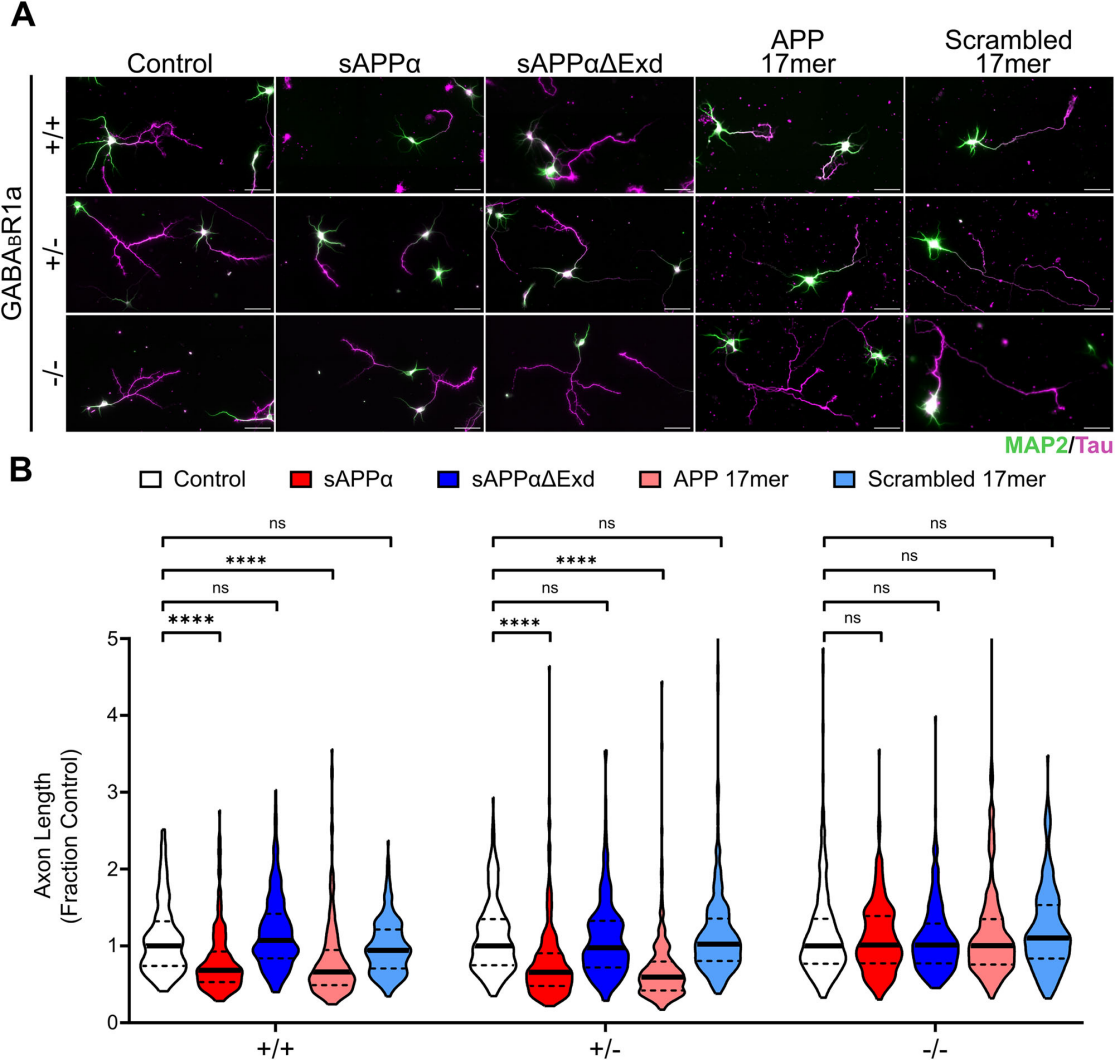
The effects of sAPPα and APP 17mer are abolished in GABA_B_R1a^−/−^ neurons. ***A***, Representative images of hippocampal primary neurons derived from wild-type (+/+), heterozygous GABA_B_R1a KO (−/+), and homozygous GABA_B_R1a KO (−/−) E18 littermates treated at DIV 0 with sAPPα, sAPPα-ΔExD, APP 17mer, or scrambled 17mer and immunostained at DIV3 with MAP2 (green, dendritic marker) and Tau (magenta, axonal marker). ***B***, Treatment with sAPPα significantly reduced axon length in both GABA_B_R1a +/+ and −/+ neurons (*N* = 60–90 neurons/trial across 3 trials; medians = 0.6613 and 0.5940, IQR = 0.5305–0.9274 and 0.4785–0.9049, respectively). Treatment with APP 17mer significantly reduced axon length in both GABA_B_R1a +/+ and −/+ neurons (*N* = 60–90 neurons/trial across 3 trials; medians = 0.6832 and 0.6568, IQR = 0.4906–0.9480 and 0.4213–0.7989, respectively). Treatment with sAPPα and APP 17mer had no significant effect on axon length in GABA_B_R1a −/− neurons. Untreated controls in 3B are the same neurons as in Figure 1*E*,*F*. Scale bars, 50 µm. Kruskal–Wallis with Dunn's multiple-comparison post hoc test were used. ****p < 0.0001; ns, not significant. Axon branch number is quantified in Extended Data Figure 3-1 and neuronal polarity is quantified in Extended Data Figure 3-2.

To test whether GABA_B_R1a is required for the inhibition of axon outgrowth by sAPPα, we cultured primary neurons from GABA_B_R1a^−/−^, GABA_B_R1a^+/−^, or GABA_B_R1a^+/+^ littermates and treated each genotype with 500 nM sAPPα, sAPPα-ΔExD, APP 17mer, and scrambled 17mer. sAPPα reduced axon length by 23% in GABA_B_R1a^+/+^ neurons and 42% in GABA_B_R1a^+/−^ neurons, whereas sAPPα-ΔExD had no significant effect on axon length (Fig. 3*A*,*B*). Similarly, 500 nM APP 17mer resulted in a 34% reduction in axon length of GABA_B_R1a^+/+^ and 36% reduction in GABA_B_R1a^+/−^ neurons compared with untreated controls, whereas scrambled 17mer had no significant effect on axon length (Fig. 3*A*,*B*). Strikingly, the effects of sAPPα and APP 17mer on axon length were abolished in GABA_B_R1a^−/−^ neurons (Fig. 3*A*,*B*). No significant effects on axon branching (Extended Data Fig. 3-1) or neuronal polarity (Extended Data Fig. 3-2) were observed across treatments in each of the three genotypes. Together, these findings demonstrate that sAPPα requires the presence of GABA_B_R1a to inhibit neurite outgrowth.

10.1523/ENEURO.0345-25.2026.f3-1 Figure 3-1 Axonal branch number is unaffected by sAPPα or APP 17mer treatment. Primary axon branch number was not significantly different with treatment of sAPPα, sAPPα-ΔExD, APP 17mer, or scrambled 17mer in any of the three genotypes (+/+),(+/-),(-/-) (N = 15-30 axons/trial across 3 trials; Untreated Control Medians = 4.5,3.0,3.0, IQRs = 2.0-7.0, 1.75-4.25, 1.0-5.0 respectively). Untreated controls in Extended Figure 3-1 are the same neurons as in Extended Figures 1-1, 1-2. Kruskal-Wallis with Dunn’s multiple comparison post hoc test were used. Comparisons not displayed are not significant (P > 0.05). Download Figure 3-1, TIF file.

10.1523/ENEURO.0345-25.2026.f3-2 Figure 3-2 Neuronal polarity is unaffected by treatment with APP peptides. The cell fraction of SA, NA, MA neurons across genotypes and treatment with APP peptides was not significantly different from WT controls (N = 90-160 neurons/trial across 3 trials; Untreated Control Means SA = 0.7167, 0.7300, 0.6700; StDev. = 0.04041, 0.1136, 0.1513; Untreated Control Means NA = 0.2267, 0.1433, 0.1159; StDev = 0.04041, 0.06658, 0.06692; Untreated Control Means MA = 0.05667, 0.1300, 0.06667; StDev = 0.01155, 0.05292, 0.05508 respectively) (N = 90-160 neurons/trial across 3 trials; Mean = 71.6; StDev. = 4%, 22.6 StDev = 4%, 5.67 StDev = 1%, respectively). Untreated controls in Extended Figure 3-2 are the same neurons as in Extended Figures 1-3. Comparisons not displayed are not significant (P > 0.05). Download Figure 3-2, TIF file.

## Discussion

Here, we found that 500 nM sAPPα reduces axon outgrowth in mouse primary hippocampal neurons (Fig. 2*D*,*F*). This effect required the presence of both GABA_B_R1a and the ExD of sAPPα, which mediates its interaction with GABA_B_R1a (Fig. 3*A*,*B*). The APP 17mer, which was previously shown to bind GABA_B_R1a and mimic the effects of sAPP on synaptic transmission (Rice et al., 2019), also reduced axon outgrowth in WT but not in GABA_B_R1a KO neurons (Fig. 3*A*,*B*). Together, these findings demonstrate that sAPPα inhibits axon outgrowth in primary mouse neurons via GABA_B_R1a.

Previously, GABA_B_R has been implicated in regulating neurite outgrowth, with activation by the agonist baclofen reported to reduce axon length and inhibition by CGP reported to enhance axon length in primary neurons (Bony et al., 2013). Our present study not only confirms these findings (Fig. 1*B*) but also establishes a role for the GABA_B_R1a isoform in this effect. We show that both partial and full ablation of GABA_B_R1a promotes neurite outgrowth (Fig. 1*D*) and the inhibition of neurite outgrowth by the GABA_B_R agonist baclofen requires the presence of GABA_B_R1a (Fig. 1*E*). Thus, the 1a isoform contributes to GABA_B_R-dependent suppression of neurite outgrowth.

Decades of research across multiple groups has demonstrated that both full length APP and sAPPα play a role in neurite outgrowth (Chau et al., 2023). The prevailing consensus is that ablation of full-length APP promotes neurite outgrowth in primary neuron cultures (Perez et al., 1997; Young-Pearse et al., 2008; Billnitzer et al., 2013; Liu et al., 2021), although a reduction in axon outgrowth has also been recently reported (Southam et al., 2019). Paradoxically, numerous studies report that application of sAPPα also promotes neurite outgrowth (Milward et al., 1992; Young-Pearse et al., 2008; Chasseigneaux et al., 2011; Billnitzer et al., 2013; Hasebe et al., 2013; Dorard et al., 2018; Liu et al., 2021). As an exception, one study found that neurons cocultured with APP KO astrocytes had elongated neurites, suggesting that sAPPα can, under certain conditions, inhibit neurite outgrowth (Perez et al., 1997). Consistent with this and aligning with the observation that APP ablation enhances neurite outgrowth, our current findings indicate that sAPPα can reduce neurite outgrowth. Our findings suggest the directionality of the effect of sAPPα may depend on its context-dependent interactions with different binding partners. We found that at low concentrations (1–200 nM), sAPPα enhances axon outgrowth, consistent with concentrations and effects reported previously (Young-Pearse et al., 2008; Billnitzer et al., 2013; Hasebe et al., 2013; Dorard et al., 2018). In contrast, at a higher concentration (500 nM), which approximates the reported K_D_ of 431 nM for binding of sAPPα to the sushi-1 of GABA_B_R1a, we observe a reduction in axon outgrowth. Importantly, neither negative controls (sAPPΔExD and scrambled 17mer) applied to wild-type neurons nor 500 nM sAPPα applied to GABA_B_R1a KO neurons reduced axon length, demonstrating that the effects observed at higher concentrations are not due to general neuronal toxicity. Rather, these findings suggest that at elevated concentrations, interaction of sAPPα to GABA_B_R1a may dominate and shift the functional outcome. APP expression is high during early development (Hung et al., 1992; Kirazov et al., 2001), providing a physiological context in which the reported effects may be observed. At lower concentrations, sAPPα may promote neurite outgrowth through binding to other interactors, such as Integrin β1 (Young-Pearse et al., 2008). Interestingly, synaptic activity is known to regulate APP processing (Cirrito et al., 2005; Cheon et al., 2008; Tampellini et al., 2009; Kleschevnikov et al., 2012b) and could thereby tightly regulate neurite outgrowth.

Our data includes multiple independent datasets examining baclofen (Fig. 1*B*,*F*) and 500 nM sAPPα (Fig. 2*D*,*F*,*B*) on wild-type neurons. These conditions were incorporated into multiple sets of experiments to allow additional treatments and genotypes to be directly compared within the same experiment. Across all experiments, both baclofen and 500 nM sAPPα produced a statistically significant decrease in axon length. However, the magnitude of these effects varied between experiments, likely reflecting inherent variability in axon lengths within and between neuronal cultures. This variability may also account for the unexpectedly larger effect size observed in the partial GABA_B_R1A KO cultures compared with the full KO cultures.

APP and GABA_B_R have each been independently implicated in neurodevelopmental disorders, including fragile X syndrome (Westmark et al., 2011, 2016a,b; Henderson et al., 2012; Ray et al., 2016; Kang et al., 2017), autism spectrum disorders (Fatemi et al., 2009; Wegiel et al., 2012; Frackowiak et al., 2013, 2020; Li et al., 2022), and Down syndrome (Best et al., 2007; Kleschevnikov et al., 2012b). In Down syndrome, APP expression is increased due to triplication (Cheon et al., 2008), and pharmacological inhibition of GABA_B_R has been shown to rescue deficits in a Down syndrome mouse model (Kleschevnikov et al., 2012a). Our findings support a functional link between dysregulation of APP and GABA_B_R signaling pathways in neurodevelopment. The ability of a short APP-derived 17mer peptide to activate this pathway highlights the therapeutic potential of targeting APP-GABA_B_R1a signaling for the treatment of neurodevelopmental disorders.

## Synthesis

Reviewing Editor: Fabienne Poulain, University of South Carolina Decisions are customarily a result of the Reviewing Editor and the peer reviewers coming together and discussing their recommendations until a consensus is reached. When revisions are invited, a fact-based synthesis statement explaining their decision and outlining what is needed to prepare a revision will be listed below. The following reviewer(s) agreed to reveal their identity: NONE.

This short manuscript analyzes the effects of soluble APPα (sAPPα) and its interaction with the gamma amino butyric acid receptor type-B subunit 1a (GABABR1A) on axon growth. Using pharmacological treatments of hippocampal neurons in culture, the authors found that application of sAPPα or of a 17 amino acid peptide derived from one of its domains reduces axon length in a GABABR1A manner. Additional experiments and quantifications now further show that the inhibitory effect of sAPPα on axon growth is not due to impaired neuronal polarization and is concentration dependent. Overall, experiments are rigorous, well described, and report a novel function of sAPPα and GABABR1A that should be of high interest for the fields of neurodevelopment and neurodegeneration. The revisions made by the authors strongly strengthen the study, which is now appropriate for publication in eNeuro. The following minor comments should be addressed:

1) In figure 1, the pictures in panel 1A are still too small to see the morphology of the neurons. The size of the pictures should be increased for visibility (the size of pictures shown in panel 1D can also be increased if the concern is to keep all pictures consistent).

2) It would be nice to show beta-actin loading controls for the Western blot shown in figure 1C, especially since the Methods indicate that beta-actin was probed for WB analyses.

3) Line 210 should indicate Figure 1D instead of Figure 1C.

4) To be consistent with Figure 1, extended Figures 1-1 and 1-2 should be combined (two panels within one extended figure). Also, since the samples analyzed in extended figure 1-1 are the same as the untreated controls in extended figures 1-2 and 3-1, why is the significant difference between control +/+ and -/- not indicated in extended figures 1-2 and 3-1?

## Author Response

Minor point 1: In figure 1, the pictures in panel 1A are still too small to see the morphology of the neurons. The size of the pictures should be increased for visibility (the size of pictures shown in panel 1D can also be increased if the concern is to keep all pictures consistent).

Response 1: We have further increased the size of panel 1A.

Minor Point 2: It would be nice to show beta-actin loading controls for the Western blot shown in figure 1C, especially since the Methods indicate that beta-actin was probed for WB analyses.

Response 2: We have included the beta-actin loading control blot. As mentioned in the methods, we loaded 8ug of primary neurons and synaptosomes but only 0.5 ug of the transfected HEK293 cells. This is the reason that there is not an apparent beta-actin band for the HEK293 lanes at this exposure. However, HEK293 cells and synaptsomes were included simply to rigorously show where the GABABR isoform should migrate on the primary neuron samples. If we loaded similar equal amounts of transfected HEK293 lysate those lanes would be overexposed and useless. We added the following text to the figure legend to hopefully clarify this for the reader to prevent confusion "Western blot of DIV 3 primary cultures probed for GABABR1 and β-actin. HEK293 cells transfected with GABABR1a and GABABR1b as well as synaptosomes are shown for reference. 8ug of protein was loaded for primary cultures and synaptosomes; whereas, only 0.5 was loaded of transfected HEK293 cells." Minor point 3: Line 210 should indicate Figure 1D instead of Figure 1C.

Response 3: This error has been corrected.

Minor Point 4: To be consistent with Figure 1, extended Figures 1-1 and 1-2 should be combined (two panels within one extended figure). Also, since the samples analyzed in extended figure 1-1 are the same as the untreated controls in extended figures 1-2 and 3-1, why is the significant difference between control +/+ and -/- not indicated in extended figures 1-2 and 3-1? Response 4: We have combined Figures 1-1 and 1-2 into Figure 1-1, panels A &B. We have also corrected figure legends and in text references to these Figures. The reason that the same untreated controls are not significant in new extended figures 1-1B and 3-1 is that the inclusion of additional comparisons in the statistical analysis reduces the statistical significance.
