# Supplementary figures and images for: sAPPα Inhibits Neurite Outgrowth in Primary Mouse Neurons via GABA B Receptor Subunit 1a
Source: eNeuro. 2026 Feb 12;13(2):ENEURO.0345-25.2026. doi: 10.1523/ENEURO.0345-25.2026 (PMC12916160; doi:10.1523/ENEURO.0345-25.2026)

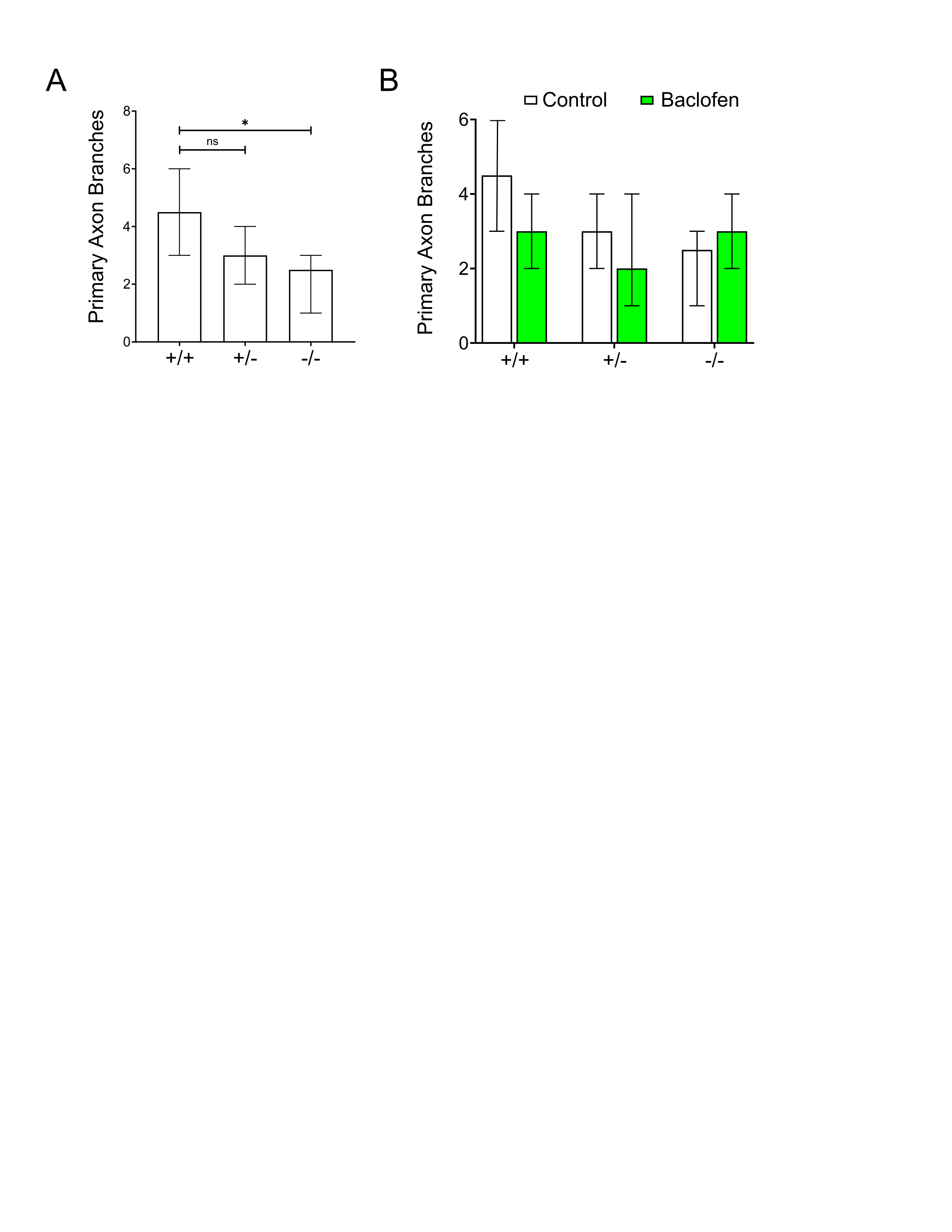

Supplement: Figure 1-1 — Full genetic ablation of GABABR1a decreases axonal branch number but is unaffected by baclofen treatment. A) The number of primary axon branches was decreased in GABABR1a KO (-/-) primary neurons (N = 15-30 neurons/trial across 3 trials; Median = 2.5, IQR = 1-5) compared to controls. Kruskal-Wallis with Dunn’s multiple comparison post hoc test were used. *P < 0.05; ns, not significant (P > 0.05). B) The number of primary axon branches of baclofen treated neurons show no significant differences from untreated controls across all genotypes (+/+),(+/-),(-/-) (N = 15-30 primary axons/trial across 3 trials; Untreated Control Medians = 4.5,3.0,3.0, IQRs = 2.0-7.0, 1.75-4.25, 1.0-5.0 respectively). Kruskal-Wallis with Dunn’s multiple comparison post hoc test were used. Comparisons not displayed are not significant (P > 0.05). Download Figure 1-1, TIF file. [file eneuro-13-ENEURO.0345-25.2026-s001.tif]

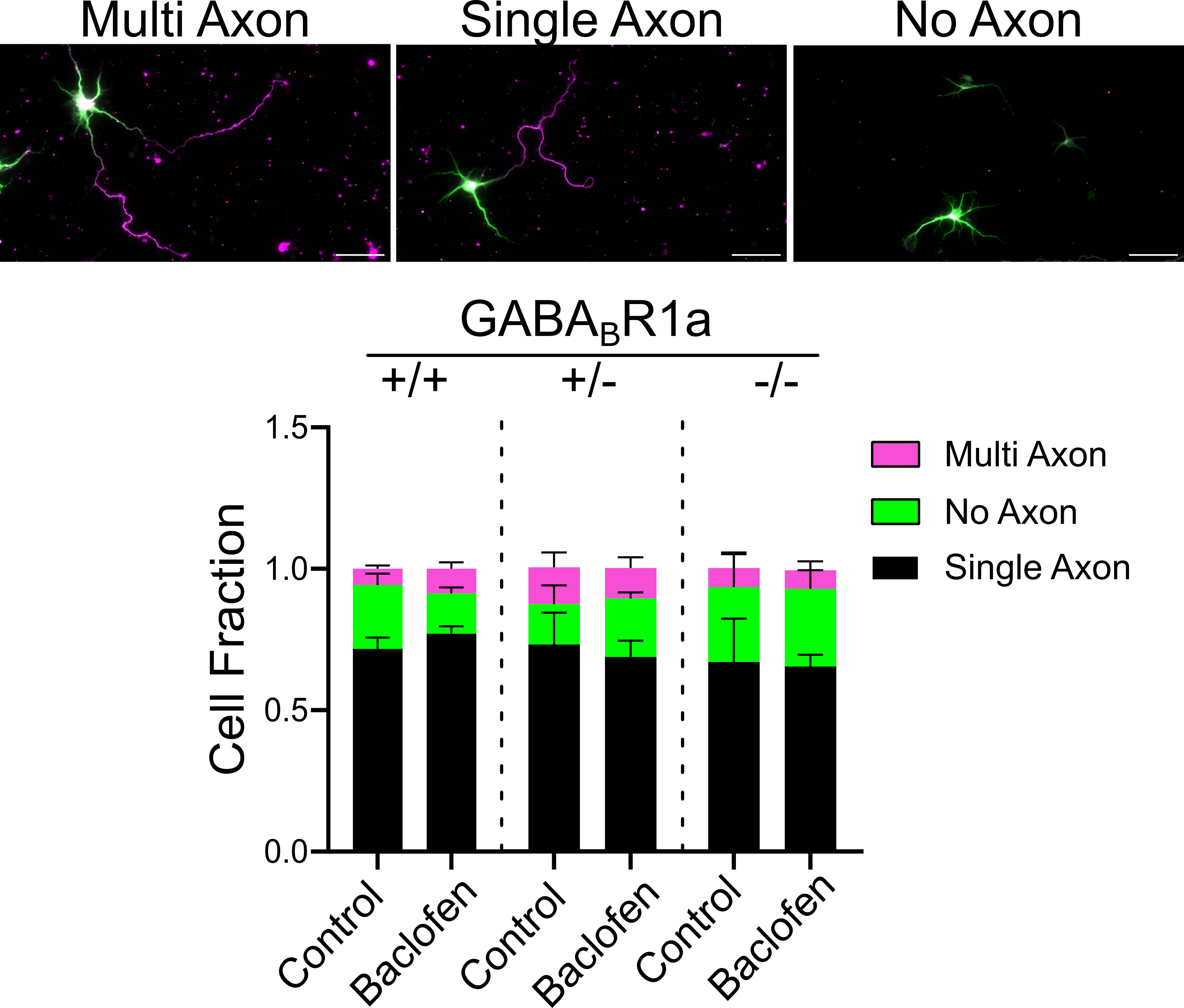

Supplement: Figure 1-2 — Neuronal polarity is unaffected by genetic ablation of GABABR1a. Representative images of neurons with multi axon (MA) single axon (SA)and no axon (NA). The fraction of SA, NA, MA neurons across genotypes all (+/+),(+/-),(-/-). Treatment with baclofen was not significantly different from untreated controls (N = 90-160 neurons/trial across 3 trials; Untreated Control Means SA = 0.7167, 0.7300, 0.6700; StDev. = 0.04041, 0.1136, 0.1513; Untreated Control Means NA = 0.2267, 0.1433, 0.1159; StDev = 0.04041, 0.06658, 0.06692; Untreated Control Means MA = 0.05667, 0.1300, 0.06667; StDev = 0.01155, 0.05292, 0.05508 respectively). Comparisons not displayed are not significant (P > 0.05). Download Figure 1-2, TIF file. [file eneuro-13-ENEURO.0345-25.2026-s002.tif]

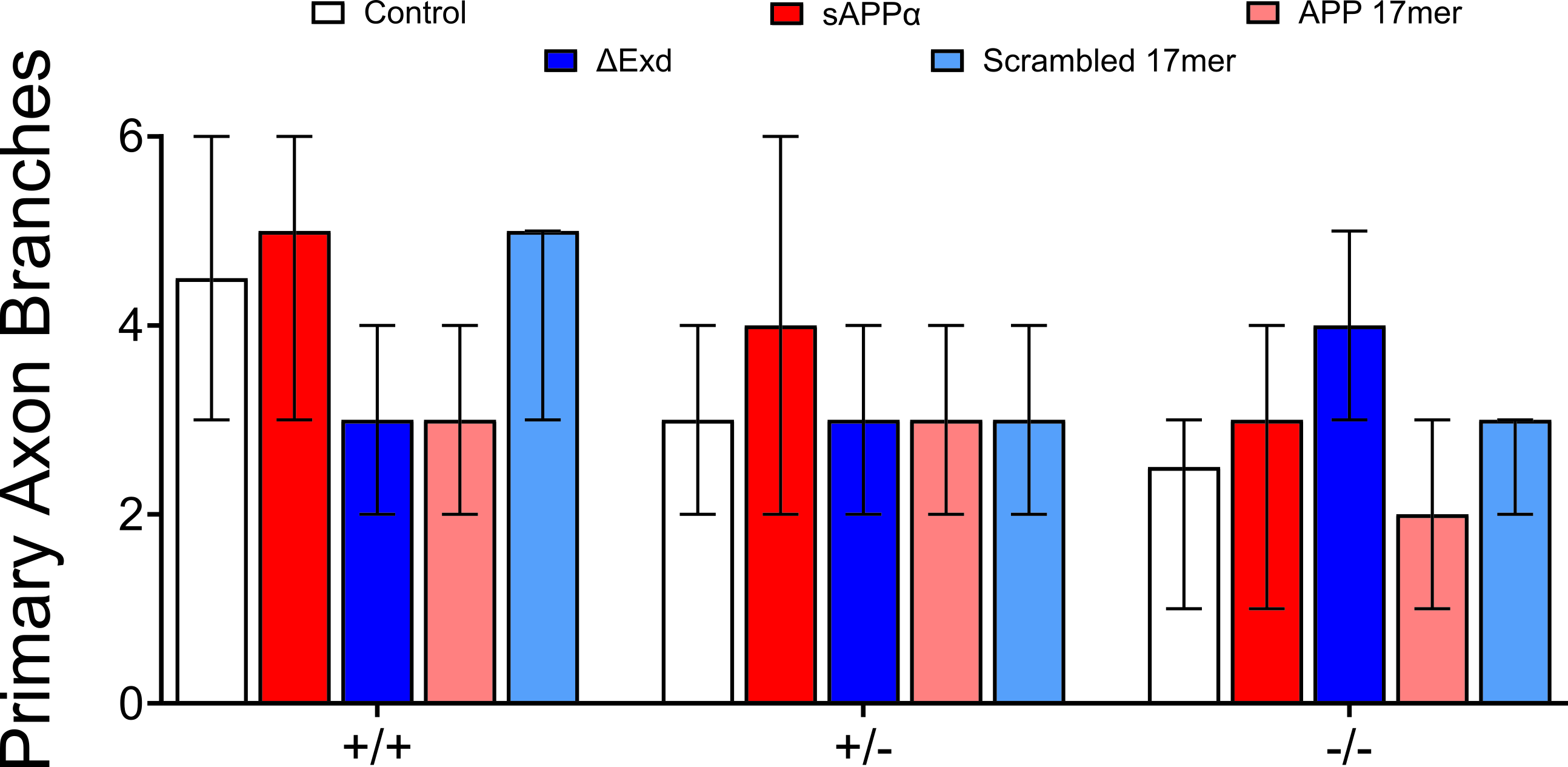

Supplement: Figure 3-1 — Axonal branch number is unaffected by sAPPα or APP 17mer treatment. Primary axon branch number was not significantly different with treatment of sAPPα, sAPPα-ΔExD, APP 17mer, or scrambled 17mer in any of the three genotypes (+/+),(+/-),(-/-) (N = 15-30 axons/trial across 3 trials; Untreated Control Medians = 4.5,3.0,3.0, IQRs = 2.0-7.0, 1.75-4.25, 1.0-5.0 respectively). Untreated controls in Extended Figure 3-1 are the same neurons as in Extended Figures 1-1, 1-2. Kruskal-Wallis with Dunn’s multiple comparison post hoc test were used. Comparisons not displayed are not significant (P > 0.05). Download Figure 3-1, TIF file. [file eneuro-13-ENEURO.0345-25.2026-s003.tif]

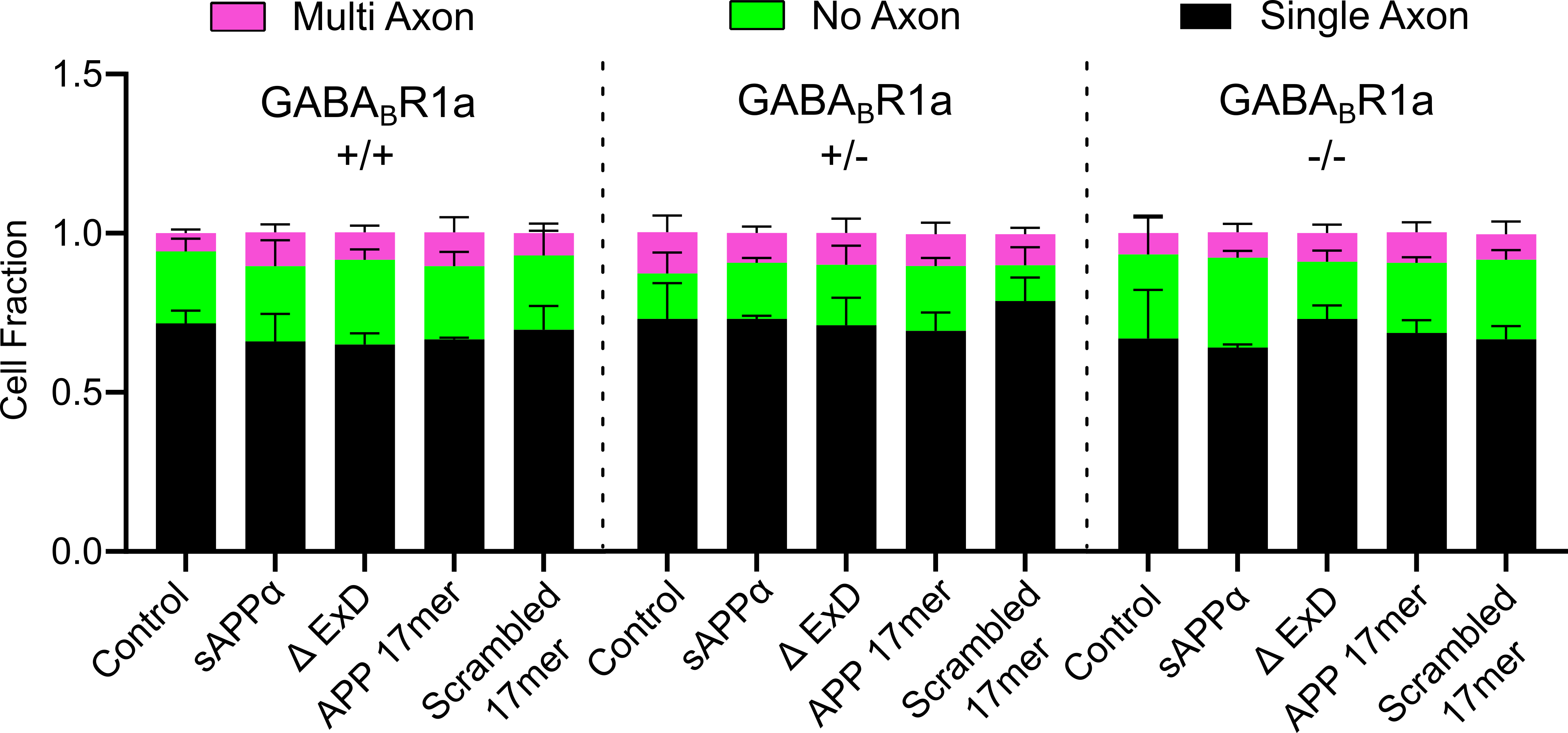

Supplement: Figure 3-2 — Neuronal polarity is unaffected by treatment with APP peptides. The cell fraction of SA, NA, MA neurons across genotypes and treatment with APP peptides was not significantly different from WT controls (N = 90-160 neurons/trial across 3 trials; Untreated Control Means SA = 0.7167, 0.7300, 0.6700; StDev. = 0.04041, 0.1136, 0.1513; Untreated Control Means NA = 0.2267, 0.1433, 0.1159; StDev = 0.04041, 0.06658, 0.06692; Untreated Control Means MA = 0.05667, 0.1300, 0.06667; StDev = 0.01155, 0.05292, 0.05508 respectively) (N = 90-160 neurons/trial across 3 trials; Mean = 71.6; StDev. = 4%, 22.6 StDev = 4%, 5.67 StDev = 1%, respectively). Untreated controls in Extended Figure 3-2 are the same neurons as in Extended Figures 1-3. Comparisons not displayed are not significant (P > 0.05). Download Figure 3-2, TIF file. [file eneuro-13-ENEURO.0345-25.2026-s004.tif]
